# The burden of skin cancer in heart transplant recipients: Impact of immunosuppressive regimens

**DOI:** 10.1016/j.jhlto.2025.100380

**Published:** 2025-08-21

**Authors:** Matteo Marro, Gabriele Roccuzzo, Erika Simonato, Gustavo Alfredo Sobrino Avellaneda, Giulia Rocco, Antonio Loforte, Mauro Rinaldi, Simone Ribero, Massimo Boffini

**Affiliations:** a Cardiac Surgery Division, Department of Surgical Sciences, City of Health and Science University Hospital, University of Turin, Turin, Italy; b Dermatology Clinic, Department of Medical Sciences, City of Health and Science University Hospital University of Turin, Turin, Italy

**Keywords:** heart transplant, immunosuppression, skin cancer, post-transplant complication, survival

## Abstract

**Background:**

Skin cancer is the most common post-transplant malignancy. We aimed to determine the incidence, timing, risk factors, and survival impact of skin cancer in heart transplant (HTx) recipients over long-term follow-up.

**Methods:**

This retrospective, single-center cohort study analyzed 568 HTx patients surviving >1 year (1990-2024). Patients with ≥1 histologically confirmed skin malignancy were compared to matched controls without skin cancer. Demographics, transplant characteristics, and immunosuppressive regimens were assessed. Logistic regression and Cox models identified independent risk factors for skin cancer and survival.

**Results:**

Of 568 eligible patients, 42 (7.4%) developed skin cancer after a median of 15.5 years. Basal cell carcinoma was most common (54.7%), followed by squamous cell carcinoma (SCC, 33.3%). Immunosuppression with calcineurin inhibitor (CNI) plus azathioprine (AZA) was independently associated with increased skin cancer risk (odds ratio [OR] 9.41, *p* = 0.044), especially for SCC (OR 6.6, *p* = 0.027). Median time to first skin tumor onset was shortest with CNI + AZA (6 years, *p* = 0.0014) compared to other AZA-free immunosuppressive regimens. Overall survival (OS) did not differ significantly between skin cancer and control groups (*p* = 0.485), but SCC was independently associated with reduced OS (HR 2.14, *p* = 0.05).

**Conclusions:**

Skin cancer is a relevant long-term complication after HTx, particularly SCC in patients receiving AZA. Our findings support limiting AZA use and reinforce the importance of structured dermatologic surveillance and early mammalian target of rapamycin conversion strategies to improve long-term outcomes.

## Graphical abstract

**Figure:**
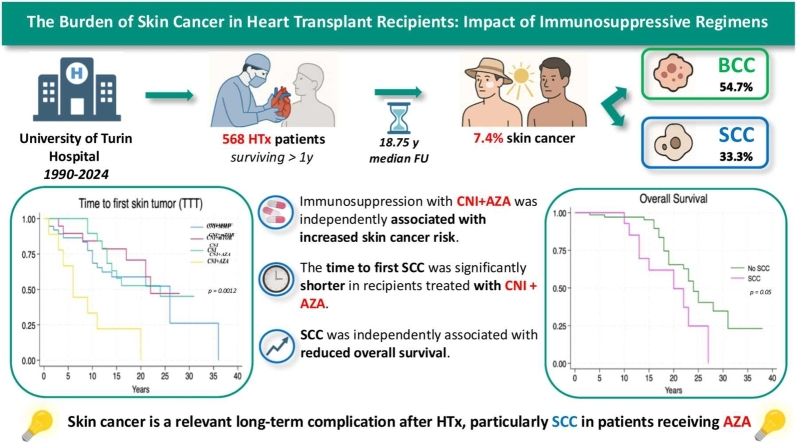

## Background

Heart transplantation (HTx) still represents the “gold standard” treatment for refractory end-stage heart failure. Despite optimal long-term results, mortality and morbidity can be affected by multiple factors. Causes of death vary over time, with the incidence of graft failure death being highest in the first 30 days and then late after transplant, infection death being most prevalent in the first year, and long-term complications such as malignancy, cardiac allograft vasculopathy, and renal failure becoming progressively more important with increasing time after transplant. In the longer term, malignancy becomes the most important cause of death and skin malignancy and lymphoma are the most common types, with skin cancer reaching nearly 20% at 10 years.^1^ Non–melanoma skin cancer (NMSC), which includes mainly cutaneous squamous cell carcinoma (SCC) and basal cell carcinoma (BCC), is the most prevalent malignancy seen in long-term immunosuppressed solid organ transplant recipients (sOTR). With a typical predominance of SCC over BCC, a more than 65-fold increased incidence of post-transplant NMSC in sOTRs has been estimated compared to the general population.^2^, ^3^ A recent meta-analysis estimated an overall skin cancer prevalence of 16%, with pooled incidence rates of 10% for SCC and 8% for BCC.^4^ The management of post-transplant NMSC is challenging due to its typically more aggressive clinical behavior compared to tumors arising in the general population.^5^ Thus, clinical decision-making and preventive strategies for both primary and secondary prevention of post-transplant NMSC rely significantly on a comprehensive understanding of the risk factors contributing to the development of multiple subsequent tumors and the dynamics of their occurrence.

The aim of this retrospective single-center observational cohort study is to determine the incidence of skin cancer in heart transplant recipients and to assess the impact of different immunosuppressive regimens on its development.

## Materials and methods

### Study population

A retrospective observational study was conducted at Città della Salute e della Scienza, University Hospital in Turin, analyzing consecutive heart transplants performed between January 1990 and December 2024. All patient information was sourced from the hospital’s database and subsequently archived within an internal computerized database. The inclusion criteria encompassed adult patients (≥18 years old) undergoing single or combined transplants (heart-lung, heart-kidney, and heart-liver) for either a first or second HTx with a follow-up of at least 12 months. A total of 757 patients were screened, of whom 568 survived the first year after transplant and continued with routine clinical follow-ups at our center. Among them, 42 patients (7.4%) developed skin cancer.

A 1:1 control group of 42 patients without a diagnosis of skin cancer was selected from the same survivor pool. Controls were matched to cases based on time since transplantation, age at transplantation, sex, underlying cardiac disease, and current follow-up duration. We analyzed baseline characteristics along with pretransplant and post-transplant data. In line with institutional and international guidelines, all recipients received long-term immunosuppressive therapy according to protocols that were revised by transplant physicians during the study period. Four main clusters of therapy immunosuppression were defined as (1) calcineurin inhibitors (CNI) plus mycophenolate mofetil (MMF), (2) CNI plus mammalian target of rapamycin (mTOR) kinase inhibitor, (3) CNI alone, and (4) CNI plus azathioprine (AZA). Diagnosis of skin malignancies was based on dermoscopic assessment by board-certified dermatologists and histopathologic examination by board-certified pathologists after surgical excision. Every effort was made to collect and validate information on any dermatologic diagnoses made outside the study center through medical records.

### Statistical analysis

Data were tested for normal distribution by Shapiro-Wilk test and a test on the equality of standard deviations (variances) on every variable was performed. Data were expressed as mean and standard deviation or median with interquartile range (IQR) 25 to 75, as appropriate. Descriptive statistics are presented as mean, median, standard deviation, and ranges for the continuous variables, and as counts and percentages for categorical variables. Mann-Whitney, chi-square with Yates corrections, and Fisher’s exact tests were used to analyze continuous and paired nominal data. Independence of observations, homoscedasticity, and absence of influential outliers were assessed before performing logistic regression analysis to identify predictors of skin cancer development. Collinearity diagnostics through variance inflation factor were used to rule out multicollinearity among independent variables. Odds ratios (ORs) with 95% confidence intervals (CIs) were computed to display the strength of association between the potential predictors and the outcomes in study. The proportional hazards assumption on the basis of Schoenfeld residuals was tested after fitting the Cox models.^6^ Study end-points included time to skin tumor (TTT), as the time from the day of transplantation to the date of the first diagnosis of any skin cancer, and overall survival (OS), as the time from day of transplantation to the date of death. For patients alive without disease tumor diagnosis and/or death event, data were censored on the date of last patient contact. Statistical difference has been considered significant for *p* < 0.05. All statistical analyses were performed using Stata/SE.v.18 Software (StataCorp, College Station, TX) and SPSS 20.0 (IBM Corp, Armonk, NY).

## Results

A total of 84 patients were included in the analysis, with 42 individuals diagnosed with skin cancer and 42 serving as controls without skin cancer. Univariate analysis revealed no significant differences between the 2 cohorts in terms of age (*p* = 0.542), body mass index (BMI) (*p* = 0.197), sex (*p* = 0.786), or underlying disease distribution (*p* = 0.921) Table 1.

**Table 1:** General Characteristics of the 2 Cohorts

	Study group (*n* = 42)	Control group (n = 42)	*p*-value
Male sex (*n*, %)	33 (78.5%)	33 (78.5%)	0.786
Age at transplant (years)	52 [43.2-56.7]	52.5 [43.2-58.7]	0.542
Age at last follow-up (years)	71.5 [64.5-79]	73.5 [60-77]	0.551
BMI	24.6±4.4	25.6±3.6	0.197
Age at first SC diagnosis (years)	65.5 [57.2-70.7]	-	
TTT (y)	15.5 [11-21]	-	
Follow-Up from HTx (years)	18.7 [13.7-23.8]	19 [15.2-23]	0.750
Immunosuppression regiment			
− CNI + MMF	17 (40.5%)	20 (47.6%)	0.6
− CNI + mTOR	7 (16.7%)	12 (28.6%)	0.5
− CNI	10 (23.8%)	9 (21.4%)	0.63
− CNI + AZA	8 (19%)	1 (2.4%)	0.04
SC type at diagnosis			
− BCC	23 (54.7%)	-	
− SCC	14 (33.3%)	-	
− Melanoma	3 (7.1%)	-	
− Other	2 (4.9%)	-	
Lesion location			
− Face	22 (52.4%)	-	
− Back	6 (14.3%)	-	
− Arm	5 (12%)	-	
− Leg	3 (7.1%)	-	
− Chest	3 (7.1%)	-	
− Abdomen	1 (2.4%)	-	
− Double location	2 (4.7%)	-	
SC relapse	8 (19.4%)	-	

Abbreviations: AZA, azathioprine; BCC, basal cell carcinoma; BMI, body mass index; CNI, calcineurin inhibitors; HTx, heart transplantation; MMF, mycophenolate mofetil; mTOR, mammalian target of rapamycin kinase inhibitor; SC, skin cancer; SCC, squamous cell carcinoma; TTT, time to skin tumor.

Among the 42 HTx patients with skin cancer, 33 (78.5%) were male. Median age at HTx was 52 years (IQR 43.25-56.75) and mean BMI of 24.6 ± 4.38. At the time of transplant, the underlying diagnosis was idiopathic cardiomyopathy in 16 patients (38%), ischemic cardiomyopathy in 18 patients (42.8%), postinfective endocarditis in 3 patients (7.1%), valvular cardiomyopathy in 3 patients (7.1%), arrhythmogenic right ventricular dysplasia and postactinic cardiomyopathy in 1 patient each (2.3%). No patient had a history of malignancy before transplantation. Median time from HTx to first diagnosis of skin cancer was 15.5 years (IQR 11-21) with a median age of 65.6 years (IQR 57.25-70.75). BCC was the first diagnosis in 23 cases (54.7%), SCC in 14 cases (33.3%), melanoma in 3 cases (7.1%), cutaneous T cell lymphoma and dermatofibrosarcoma protuberans in 1 case each (2.3%), and a diagnosis of Merkel cell carcinoma was confirmed in a patient with previous SCC diagnosis as first skin cancer.

The lesion was located on the face in 22 cases (52.4%), the back in 6 cases (14.3%), the arms in 5 cases (12%), the legs in 3 cases (7.1%), the chest in 3 cases (7.1%), and the abdomen in 1 case (2.4%). Additionally, involvement of both the face and chest was observed in 1 case (2.4%), while both the back and chest were affected in 1 case (2.4%). At the time of diagnosis, the immunosuppression regimen was composed of CNI plus MMF in 17 cases (40.5%), CNI plus mTOR in 7 cases (16.7%), CNI alone in 10 cases (23.8%), and CNI plus AZA in 8 cases (19%). Surgery was the primary treatment in 95.2% of cases, while radiotherapy in 4.8% of cases. After the first diagnosis, 9 (25.7%) patients were switched to immunosuppression regimen with mTOR. A second diagnosis of skin cancer was observed in 8 patients (19.4%), with 6 cases of BCC and 2 cases of SCC. The median follow-up from HTx was 18.75 years (IQR 13.7-23.8). Nineteen patients (45.23%) died after a median follow-up of 18.9 years (IQR 14.8-21.1), with a median age at the time of death of 76.1 years (IQR 70-80.6). Among these, 18 deaths were attributable to the natural course of long-term transplantation, and 1 was due to skin cancer (Merkel cell carcinoma).

Logistic regression analysis showed that age (OR = 0.98, 95% CI: 0.95-1.03, *p* = 0.542), BMI (OR = 0.93, 95% CI: 0.83-1.04, *p* = 0.197), and male sex (OR = 0.86, 95% CI: 0.30-2.51, *p* = 0.786) were not significantly associated with the development of any skin tumor. Similarly, underlying disease status did not demonstrate a significant impact on tumor occurrence (*p* = 0.921). However, the immunosuppression regimen was significantly associated with tumor development (*p* = 0.0498). Patients treated with CNI + AZA exhibited a markedly increased risk of skin cancer (OR = 9.41, 95% CI: 1.07-83.02, *p* = 0.044). While for BCC no significant associations were observed with age (*p* = 0.691), BMI (*p* = 0.894), sex (*p* = 0.834), disease (*p* = 0.8796), or immunosuppression regimens (*p* = 0.8803), SCC development was significantly associated with immunosuppression with CNI + AZA (OR = 6.6, 95% CI: 1.24-35.23, *p* = 0.027; Fisher test *p* = 0.03), despite no significant association with age (*p* = 0.154), BMI (*p* = 0.167), sex (*p* = 0.547), or disease (*p* = 0.8807).

**Figure 1:**
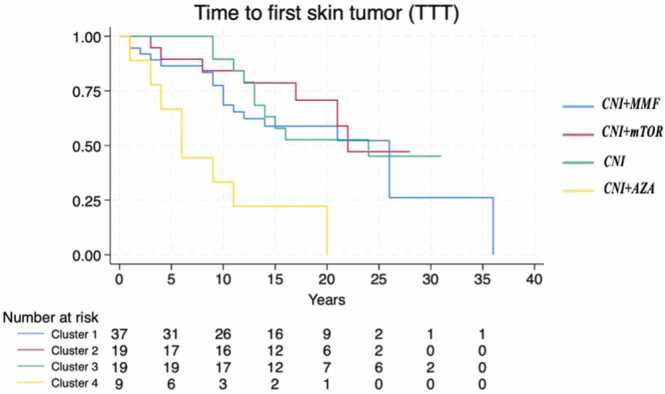
Time from transplantation to first tumor diagnosis (TTT) according to different immunosuppression regimens. Cluster 1: CNI + MMF, Cluster 2: CNI + mTOR, Cluster 3: CNI, Cluster 4: CNI + AZA. AZA, azathioprine; CNI, calcineurin inhibitors; MMF, mycophenolate mofetil; mTOR, mammalian target of rapamycin.

**Figure 2:**
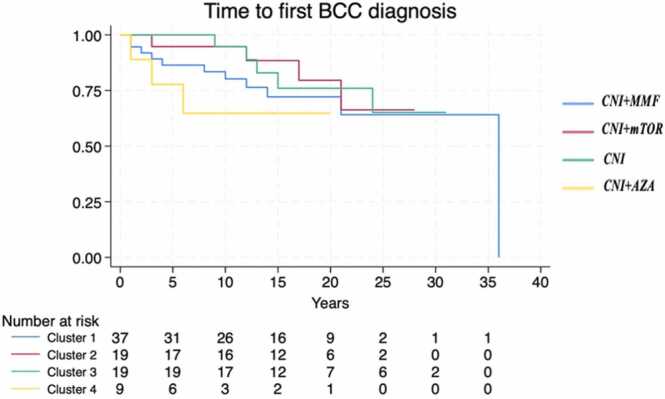
Time from transplantation to first BCC diagnosis according to different immunosuppression regimens. Cluster 1: CNI + MMF, Cluster 2: CNI + mTOR, Cluster 3: CNI, Cluster 4: CNI + AZA. AZA, azathioprine; BCC, basal cell carcinoma; CNI, calcineurin inhibitors; MMF, mycophenolate mofetil; mTOR, mammalian target of rapamycin.

**Figure 3:**
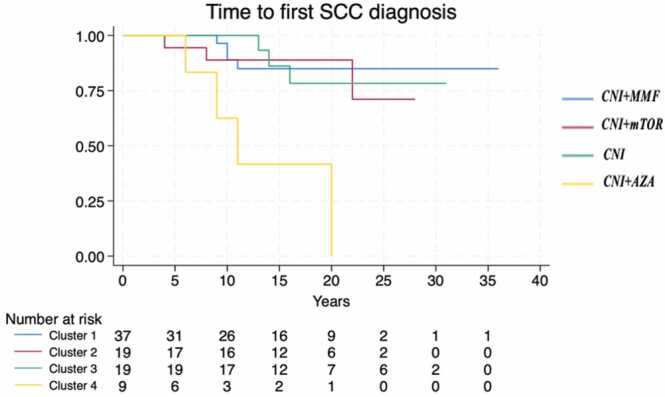
Time from transplantation to first SCC diagnosis according to different immunosuppression regimens. Cluster 1: CNI + MMF, Cluster 2: CNI + mTOR, Cluster 3: CNI, Cluster 4: CNI + AZA. AZA, azathioprine; CNI, calcineurin inhibitors; MMF, mycophenolate mofetil; mTOR, mammalian target of rapamycin; SCC, squamous cell carcinoma.

**Figure 4:**
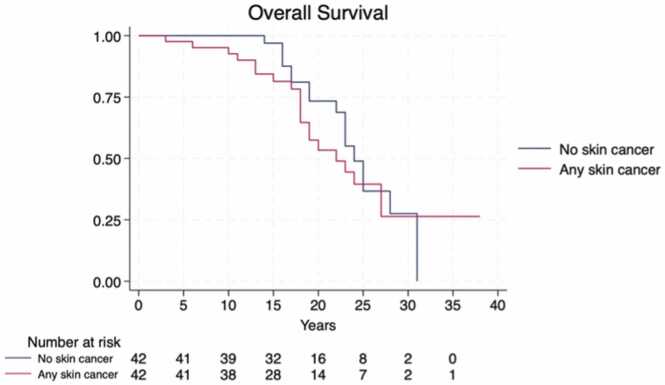
Overall survival of HTx patients with and without skin cancer. HTx, heart transplant.

**Figure 5:**
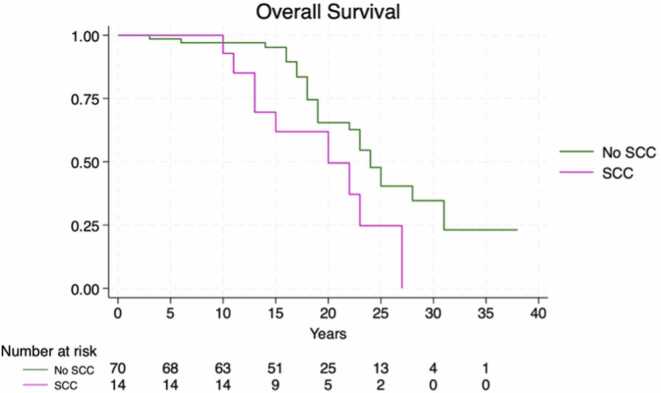
The impact of SCC development on overall survival, adjusted for immunosuppression cluster. SCC, squamous cell carcinoma.

The median TTT varied significantly by immunosuppression regimens (*p* = 0.0012), with the association CNI + AZA demonstrating the shortest time to event (median: 6 years, 95% CI: 1-NA), Figure 1. The time to first BCC did not significantly differ across clusters (*p* = 0.385), Figure 2, while the time to first SCC was significantly shorter in recipients treated with CNI + AZA (*p* = 0.0014), Figure 3. The OS did not differ significantly between patients with and without skin cancer (median OS: 22 vs 24 years, *p* = 0.485), Figure 4. However, when analyzing the clusters of tumors independently, SCC development was associated with reduced survival (median OS: 20 vs 24 years, *p* = 0.02), confirming its negative prognostic role for reduced OS after adjusting for immunosuppression regimen (Hazard Ratio = 2.14, 95% CI: 1.00-4.57, *p* = 0.05), Figure 5.

## Discussion

HTx remains the gold-standard therapy for patients with end-stage heart failure refractory to medical and device-based therapies. Advances in surgical techniques, immunosuppressive regimens, and post-transplant management have significantly improved survival rates and quality of life.^7^ However, long-term outcomes are influenced by several factors, including rejection, infection, malignancy, and allograft vasculopathy. While short-term survival following HTx has improved significantly, long-term complications remain a challenge. Malignancies represent a significant long-term complication of HTx, primarily due to the lifelong immunosuppressive therapy required to prevent graft rejection. The increased incidence of cancer in transplant recipients is well documented, and skin cancer represents the most common malignancy in the adult heart transplant population.^8^

Our study, based on high–quality registry-based medical charts, reports an incidence lower than that documented in some previous literature.^4^ It includes 84 patients with an extensive follow-up period demonstrating an increased risk of skin cancer among patients treated with AZA, particularly for SCC. Furthermore, the time from HTx to first diagnosis of skin cancer is shorter in patients treated with AZA, with this finding being statistically significant specifically for SCC. Our findings are consistent with those reported in the literature and previously exposed. A systematic review and meta-analysis by Jiyad et al^9^ showed a significantly increased risk of SCC in relation to AZA exposure and no significant associations between AZA treatment and BCC. The association between SCC and reduced OS in our cohort, despite rare skin cancer–related deaths, may reflect SCC acting as a surrogate marker of cumulative immunosuppressive burden, frailty, and comorbidities in long-term survivors. These factors may predispose to competing causes of death, including other malignancies, infections, and cardiovascular events, thereby contributing to the observed mortality signal.

Today, our immunosuppression protocol consists of induction therapy by rabbit antithymocyte globulin for 3 to 5 days according to immunological cross-match, intravenous glucocorticoids starting from the OR and lasting 3 days, and a maintenance immunosuppressive therapy employing a 3-drug regimen consisting of a CNI, an antimetabolite agent, and oral glucocorticoids, these discontinued after the first year in the absence of a significant cellular rejection history.

The administration of AZA in our patient cohort corresponds to the initial era of transplantation (1990-2005) and an earlier immunosuppressive protocol adoption nowadays is not adopted, highlighting the extended follow-up duration of our study population. Currently, the standard maintenance immunosuppressive regimen for HTx recipients primarily consists of tacrolimus, MMF, and corticosteroids, as documented by the Scientific Registry of Transplant Recipients database.^10^

The International Society for Heart & Lung Transplantation (ISHLT) recommended the administration of CNI + MMF as primary regimens, while AZA may be considered for inclusion in the immunosuppressive regimen in patients who do not tolerate other therapies such as MMF or mTOR.^11^ Several well-designed clinical trials have compared the MMF and AZA in HTx, highlighting the superiority of the first in terms of cellular rejection and survival.^12^, ^13^ According to the literature, it is possible that switching to or adding a mTOR-based immunosuppressive therapy to a patient’s regimen may be beneficial in presence of malignancy.^11^ However, despite these antitumor effects, mTOR inhibitors are rarely given right after solid organ transplantation, because they have been associated with the increased incidence of wound healing complications and severe side effects, including peripheral edema and hypertriglyceridemia.^14^

Understanding the risk factors, early detection strategies, and management approaches is crucial for improving long-term survival and quality of life in HTx recipients.^15^ Thus, a consensus statement for timing of initial skin cancer screening in adult sOTR was developed.^2^ Several factors contribute to the heightened risk of malignancy in HTx recipients. First of all, chronic exposure to ultraviolet (UV) radiation, particularly UVB, is considered the key carcinogenic factor in NMSC development. This is supported by the observation that lesions predominantly arise on sun-exposed areas of the skin and are more prevalent among individuals residing in regions with high levels of solar radiation.^9^, ^16^, ^17^ The UV–induced gene mutations, found in SCC, commonly occur in sun-exposed skin, indicating that immunosurveillance is crucial to prevent these mutated cells from undergoing malignant transformation. On the other hand, the impact of immunosuppressive therapy on the development of NMSC is well established.^18^, ^19^, ^20^, ^21^ These agents contribute to tumor development and progression through multiple mechanisms, including the acceleration of tumor growth, the suppression of tumor immunosurveillance, the higher susceptibility to UV–induced DNA damage, and the activation of proto-oncogenes.^12^, ^13^, ^22^ Notably, this association appears to be influenced by the specific class of immunosuppressive medications. Chronic immunosuppression with CNIs and AZA has been associated with higher cancer risk due to their suppression of tumor surveillance mechanisms.^23^, ^24^, ^25^

Antimetabolites that inhibit purine synthesis, such as AZA and mycophenolic acid as well as antifolate drugs (methotrexate), inhibit DNA-damage repair, which could accelerate tumorigenesis by leaving UV-light–associated DNA damage unrepaired. This direct mutagenic effect significantly increases the risk of SCC, making UVA protection critical for patients on AZA therapy.^24^, ^26^

Instead, mTOR inhibitors, such as sirolimus and everolimus, may offer some protective effects against malignancy by their antiproliferative and antineoplastic mechanism.^27^ Rivinius et al^28^ in a retrospective analysis showed that treatment with mTOR >1 year was associated with a lower cutaneous malignancy recurrence at 2 and 5 years after the initial diagnosis. Asleh et al^29^ reported in a large cohort that sirolimus-based immunosuppression without CNI was associated with significantly decreased risk of subsequent NMSC occurrence.

In addition, Nair et al^30^ identified additional recipient–related risk factors for skin cancer through an analysis of the United Network for Organ Sharing (UNOS) dataset, including male sex, White ethnicity, older age, malignancy at the time of listing or transplantation, and thymoglobulin induction, which were found to be major contributors. Same results have been sought by analyzing the Scientific Registry of Transplant Recipients database.^31^

The lower incidence of skin cancer–related mortality observed in our study might be attributed to the intensive and well-structured follow-up schedule, which includes twice weekly/monthly on-site visits during the first year, bimonthly visits in the second year, quarterly visits in the third year, every 4 months from the fourth year onward, and biannual visits from the fifth year, provided no adverse events occur. As suggested by the ISHLT guidelines, HTx recipients should have close skin cancer surveillance, including education on preventive measures and yearly dermatological exams.^2^, ^11^, ^19^

Our study suffers from both conceptual and methodological limitations: first, the retrospective nature of the study itself. Moreover, no data regarding previous sun exposure and photo-protective measures were available at the time of data collection. However, the key strength of our analysis lies in the large patient cohort and the extended follow-up period, which, to our knowledge, has no published equivalent to date. Several studies have investigated this topic, primarily focusing on kidney transplant recipients, with smaller patient populations and shorter follow-up periods.^3^, ^32^, ^33^, ^34^, ^35^

## Conclusions

HTx patients appear to be at increased risk for developing multiple skin cancers, some of which may demonstrate aggressive behavior and contribute to morbidity and mortality. In our cohort, we observed an association between AZA use and SCC development. The intensive follow-up in our program may facilitate earlier diagnosis, timely treatment, and potentially reduced mortality, although causality cannot be established from our data. Future studies, ideally prospective, are needed to confirm these findings and to evaluate whether newer immunosuppressants with a better safety profile, photo–protective systemic drugs, and treatments for precancerous lesions—together with improved patient education on sun protection—can effectively reduce the incidence of skin cancers in this high-risk population.

## Disclosure statement

The authors declare that they have no known competing financial interests or personal relationships that could have appeared to influence the work reported in this paper.

We thank the multidisciplinary transplant team at AOU Città della Salute e della Scienza—University of Turin Hospital—for their commitment to excellence in patient care and data collection.

The authors report no relevant financial relationships or conflicts of interest with respect to the research, authorship, and/or publication of this article.
